# Industrializing Autologous Adoptive Immunotherapies: Manufacturing Advances and Challenges

**DOI:** 10.3389/fmed.2018.00150

**Published:** 2018-05-23

**Authors:** Rohin K. Iyer, Paul A. Bowles, Howard Kim, Aaron Dulgar-Tulloch

**Affiliations:** ^1^Centre for Advanced Therapeutic Cell Technologies, Toronto, ON, Canada; ^2^General Electric Healthcare, Cell and Gene Therapy, Marlborough, MA, United States; ^3^Centre for Commercialization of Regenerative Medicine, Toronto, ON, Canada

**Keywords:** autologous, cellular therapy, chimeric antigen receptor, bioreactor, scale-out, CAR T-cells, NK cell, immunotherapy manufacturing

## Abstract

Cell therapy has proven to be a burgeoning field of investigation, evidenced by hundreds of clinical trials being conducted worldwide across a variety of cell types and indications. Many cell therapies have been shown to be efficacious in humans, such as modified T-cells and natural killer (NK) cells. Adoptive immunotherapy has shown the most promise in recent years, with particular emphasis on autologous cell sources. Chimeric Antigen Receptor (CAR)-based T-cell therapy targeting CD19-expressing B-cell leukemias has shown remarkable efficacy and reproducibility in numerous clinical trials. Recent marketing approval of Novartis' Kymriah™ (tisagenlecleucel) and Gilead/Kite's Yescarta™ (axicabtagene ciloleucel) by the FDA further underscores both the promise and legwork to be done if manufacturing processes are to become widely accessible. Further work is needed to standardize, automate, close, and scale production to bring down costs and democratize these and other cell therapies. Given the multiple processing steps involved, commercial-scale manufacturing of these therapies necessitates tighter control over process parameters. This focused review highlights some of the most recent advances used in the manufacturing of therapeutic immune cells, with a focus on T-cells. We summarize key unit operations and pain points around current manufacturing solutions. We also review emerging technologies, approaches and reagents used in cell isolation, activation, transduction, expansion, in-process analytics, harvest, cryopreservation and thaw, and conclude with a forward-look at future directions in the manufacture of adoptive immunotherapies.

## Introduction

Adoptive cellular immunotherapies are an exciting and paradigm-shifting modality for cancer treatment, highlighted by the recent market approval of two chimeric antigen receptor (CAR) T cell products by the US Food and Drug Administration (FDA): Kymriah (tisagenlecleucel) from Novartis for pediatric and young adult patients with ALL as well as adult patients with large B-cell lymphoma including DLBCL, and Yescarta (axicabtagene ciloleucel) from Gilead/Kite Pharma for adult DLBCL. These therapies are demonstrating remarkable success in clinical trials; Kymriah (CTL019), for example, has shown an astonishing 83% complete remission rate in clinical trials (See Kymriah product insert; https://www.pharma.us.novartis.com/sites/www.pharma.us.novartis.com/files/kymriah.pdf).

These therapies harness a patient's own cells, genetically engineered to confer novel targeting and activation properties, to direct immune responses toward cancer cells. According to clinicaltrials.gov as of November 2017, there are currently more than 260 active, recruiting or completed worldwide trials utilizing CAR-T cells; 92 trials utilizing T-cell Receptor (TCR)-modified T cells, and 121 trials utilizing donor-matched natural killer (NK) cell therapies for treatment of various hematological and solid tumor cancers, which hold promise to potentially cure where current standard treatments of chemotherapy, radiation therapy, and surgery prove unsuccessful.

As these therapies mature from treating tens to hundreds of patients during clinical trials to tens to hundreds of thousands of patients post regulatory approval, significant manufacturing challenges remain to be overcome if these therapies are to be manufactured for the global cancer population in a cost-effective, reproducible, and efficient manner. A number of these protocols still rely on discrete open and manual processing steps across the workflow, which are heavily susceptible to operator-to-operator variability, contamination, and are not amenable to scale-out.

**Figure 1:**
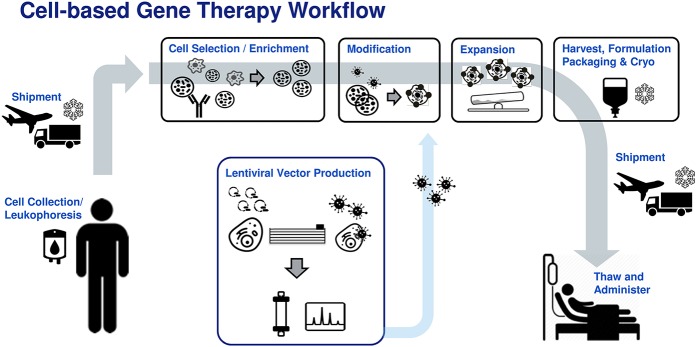
Generic patient specific adoptive immunotherapy workflow. Donor leukophoresis sample undergoes selection to enrich for the cell of interest (e.g., T-cells, NK cells). Previously manufactured viral vector is added to genetically modify the immune cells, conferring new cancer recognition receptors. The modified cells are then expanded to therapeutically relevant numbers, followed by washing, formulation, bagging, and cryostorage into an infusible patient dose. The cell product is shipped to the clinical site where it is thawed and administered to the patient.

There is now a more concerted effort among manufacturers to utilize traditional bioprocess principles to close, automate, and control these processes to ensure critical quality attributes (CQA) of the cell product are consistently maintained and manufacturing processes are cost-effective and risk-mitigated (1–5). These solutions must be developed in-hand with the tool and reagent providers to meet the unique needs of cell therapy manufacturing, where donor source materials are limited and highly variable. This focused review will look at current solutions across a typical autologous or patient-matched manufacturing workflow (Figure 1) and highlight remaining challenges toward industrialization of these processes.

## Current state and future perspectives on immunotherapy manufacturing

### Reagents for cell therapy manufacture

Traditionally, immune cell expansion in research environments has relied on the use of animal or human sera, which has translated into the inclusion of these relatively undefined reagents in clinical cell manufacturing protocols. This is potentially problematic as the use of serum introduces the possibility of (a) greater process variability due to lot-to-lot differences (6, 7) and (b) safety risks to the patient (8). Further, as patient demand for these new cell therapies increases, a sufficient and robust supply of quality sera will become challenging to secure.

Manufacturing of cell therapy products is now possible with serum-free (SF) or xenogeneic-free (XF) media and reagents. Still, many reagents are not fully defined today due to the use of undefined additives (such as human serum) or other blood-derived factors such as fibroblast growth factor (FGF). Animal component free (ACF) media that are chemically defined (CD) meet the stricter definition of not containing any human or non-human animal components, and being mixed in defined or known quantities. With the advent of both eukaryotic and prokaryotic recombinant expression systems, the availability of ACF media has become more commonplace in recent times. Still, it is critical to establish back-up suppliers for raw materials to avoid disruptions to supply chain during cell therapy manufacture.

Another important requirement for media manufacturing, particularly for later clinical stage and commercial use, is that the reagents comply with good manufacturing practices (GMP) standards. Adherence to GMP standards ensures a high degree of quality in production as well as control (9). While the use of GMP grade reagents and ACF media passes a higher level of scrutiny in terms of compliance with clinical requirements, there is also a higher cost associated with the sourcing of these reagents, often requiring custom batches for niche reagents or products. An equally important consideration is packaging of media in 0.5–1 L bottles, requiring open manipulation in BSCs. The adoption of bags with sterile weldable inputs and outputs to enable direct connections with manufacturing systems will be a key step toward industrialization and conformance with GMP workflows. As demand increases and economies of scale are attained, the relative cost of these GMP reagents should also decrease.

### Selection

In autologous immunotherapies, the starting material is typically collected through leukapheresis, where the leukocytes are separated out and the remaining blood products are returned to the patient. As there is inherent variability in the cell populations in these leukapheresis products, processes to remove unwanted cells or isolate specific populations of cells have been developed using a variety of technologies including physical separation via centrifugation, magnetic, fluorescent, as well as acoustic-based selection.

Cells types can be separated based on size through centrifugation, with or without the use of density gradient media systems (such as Ficoll, for example), which enables removal of unwanted fractions of leukapheresis product such as granulocytes, platelets and remaining red blood cell contaminants. This technology is well-established and there are now several closed and automated systems available to separate out undesired cells using density gradient separation such as the Sepax II (GE Healthcare, formerly Biosafe) and the Elutra (Terumo BCT) (10). However, the principles upon which they operate rely on inherent differences in cell size between these fractions, thus making them unable to discriminate between diverse cell phenotypes with similar sizes (for example, healthy T cells vs. leukemia cells).

Antibodies can be used to isolate cells based on cell surface marker expression. These antibodies can be conjugated to fluorochromes, magnetic beads, and agarose-coated beads. Fluorescence-Activated Cell Sorting (FACS) is a common technology used in research environments for separation of cells and can isolate sub populations by tagging of multiple surface antigens (for example, CD4+/CD25+ (54)). One of the main challenges with FACS is that the throughput and recoveries are relatively low compared to other systems, and a typical run may be very time consuming for sorting of rare populations or large starting doses. As most cell sorters are droplet based, they are also open to environmental and other extrinsic sources of contamination. To address the need for system closure during flow-based sorting, the WOLF Cell Sorter (Nanocollect) and MACsQuant Tyto (Miltenyi Biotec) use disposable cartridges, with a sorting rate of up to 30,000 cellular events per second (MACsQuant), however even at the maximum rate it would still take several hours to process a full leukapheresis product.

Magnetic-Activated Cell Sorting (MACS) using antibodies conjugated to magnetic beads is currently the most common method for isolating a population of cells, as it is quicker and has greater scalability than FACS. Miltenyi Biotec and ThermoFisher have developed closed system magnetic cell selection technologies (CliniMACS Prodigy and CTS Dynamag, respectively) along with corresponding reagents that enable isolation of specific cell populations (11, 12). Both magnetic-based selection technologies, however, lack the ability to release the magnetic beads from the isolated cell populations, which may be undesirable for immunotherapies prior to infusion into the patient. STEMCELL Technologies has developed technology that enables the release of the magnetic beads from the isolated cells (EasySep™). This technology currently is only available as a research use only (RUO) product, but there are plans to make a GMP version under license to GE Healthcare. Juno Therapeutics' acquisition of Stage Cell Therapeutics gives them access to Stage Cell's “streptamer” technologies which allows isolation through reversible binding of antibodies to magnetic or agarose beads, enabling selection through MACS or agarose-based chromatography columns (13). These latter examples illustrate how industry collaboration has helped to advance the field of cell selection.

Novel technologies that could be used in the future for cell selection include FloDesign Sonics' Acoustic Separation Technology, which isolates cells of interest based on varied sizes of antigen-bead combinations in a closed, GMP-compliant manner; SonoSep's Acoustic Wave Separation Technology, capable of fluid-particle and fluid-fluid separation based on ultrasonic standing waves; and Buoyancy-Activated Cell Separation (BACS) from Cesca Therapeutics, which uses antibodies attached to microbubbles to isolate cells of interest (14). As knowledge of the properties of immune cells improves and tunable conjugation chemistry evolves, it is anticipated that greater control and specificity of antibody-based selection will also evolve, bringing costs down, and reducing processing time and complexity for the operator.

### Activation

There are several technologies available for the activation of immune cells, including cell-based activation, bead-based activation, and antibody-based activation. Antigen Presenting Cells (APC) such as dendritic cells (DCs) and macrophages are endogenous activators of T cells. APCs are used in the activation of tumor infiltrating lymphocytes (TILs) (15) and viral-specific T cells (16). The benefit of using APCs for activation in manufacturing of cells *ex vivo* is that they provide a more *in vivo*-like stimulation of immune cells. There are several challenges with using APCs that include a) the cost of generating GMP-qualified APCs, b) risks of incomplete removal from the end therapeutic cell population c) the potential donor-to-donor variation in DCs'/monocytes' ability to activate specific T cell populations, and d) the limiting amount of these activating cells present in source material, particularly if using autologous feeder cells from critically ill patients.

Artificial Antigen Presenting Cells (aAPC) are genetically engineered cell lines that constitutively express antigens that drive the activation and expansion of specific cell types in a more controlled way than APCs. Artificial APCs have been particularly effective in the expansion of NK cells where the K562 cell, for example, has been genetically modified to express membrane bound IL-15 and 4-1BBL, yielding over 1,000-fold expansion of NK cells after 3 weeks of culture (17). Challenges in using aAPCs in immunotherapies include the time and cost in engineering, expanding, and qualifying the aAPC lines, as well as the cost and risk of their continued production.

Bead-based activation reagents are the most common activation reagent in commercial immunotherapy manufacturing of cell therapies since they produce consistent activation and have led to simplified manufacturing workflows. Dynabeads CD3/CD28 (ThermoFisher) use magnetic beads linked to anti-CD3 and anti-CD28 antibodies for activation (18, 19). Although these beads produce robust expansion, removal of magnetic beads before infusion into the patient remains challenging, and can additionally result in loss of final cellular product. Miltenyi Biotec's T cell Activation/Expansion kits use biotinylated antibodies against CD3, CD28, and CD2 that can be linked to MACSiBead 50-nm superparamagnetic particles, however this product is currently not available as a GMP product.

Several non-magnetic T cell activation reagents have been developed to reduce the complexity of the manufacturing workflow, mainly to reduce the need for removal of the magnetic beads at the end of culture. Miltenyi Biotec's MACS GMP TransAct CD3/CD28 beads are a colloidal polymeric nanomatrix covalently attached to humanized recombinant agonists of human CD3 and CD28 (11). As the beads have a lower molecular weight than cells, they can be removed from the final product through centrifugation. STEMCELL Technologies' Immunocult T Cell Activators are tetrameric antibody complexes based on crosslinking of CD3, CD28, and CD2 cell surface ligands via a central linker domain (20). As with Miltenyi Biotec's TransAct beads, the Immunocult T-cell Activator can be removed through centrifugation. Currently, Immunocult T Cell Activators are only available as RUO product, however there are plans to make them a GMP-compliant reagent with GE Healthcare. Juno Therapeutics' Expamer technology uses a complex of 5–10 Streptamers that can bind CD3/TCR complex and its co-stimulatory molecule, CD28. The benefit of these Expamers is that they are easily removed through centrifugation or perfusion at the end of the culture.

### Transduction

Transduction describes the step where gene modification (i.e., addition of CAR or TCR) occurs via introduction of an integrating viral vector, typically gamma-retroviral (gamma-RV) or lentiviral (LV), to the target cells. Transduction can be performed during T-cell activation or the subsequent 1–3 days, with the latter offering higher efficiencies due to the increased proportion of actively dividing cells (21). The process itself is usually a simple addition of the vector reagent to the culture vessel. This is preferably done in a closed manner. Indeed, good transduction efficiencies have also been demonstrated in the CliniMACS prodigy (11, 22) which incorporates programs and a flow path to accommodate this step.

Good transduction efficiencies rely on increasing the probability of cell-vector particle interactions. A common parameter used is multiplicity of infection (MOI), defined as the number of functional vector particles per target cell. However, absolute vector concentration may be a more meaningful parameter than MOI, particularly when working with processes that have low or variable cell densities at the time of vector introduction (23). Chemical enhancers including cationic polymers (e.g., polybrene, DEAE-dextran) or peptides (Retronectin, Vectofusin-1) can also improve vector-target cell interactions to improve transduction efficiencies. Polybrene is often used in research settings, particularly in the context of vector titering, but does carry concerns of toxicity and thus is not often used in clinical protocols. Retronectin has been traditionally used in gamma-RV protocols, but requires manual pre-coating of culture vessels. Physical methods to increase cell-vector interaction such as spinoculation have also demonstrated improved efficiencies or lower vector concentration requirements (24).

Other factors that impact transduction include the design and quality of the vector itself, particularly as it relates to the choice of viral envelope protein which is the primary determinant of vector tropism. The most common lentiviral vectors are based on the VSVg envelope glycoprotein. VSVg binds to low density lipoprotein receptor (LDLr), which is broadly expressed in most human cells (25). Importantly, Amiranche et al. showed that LDLr is lowly expressed in unstimulated T cells and only upregulated upon activation (26), explaining low transduction of LV in unstimulated T cells. Alternative envelope glycoproteins such as RD114 (27, 28), Baboon retroviral envelope glycoprotein (29) and Measles Virus envelope protein (30) are actively being explored as alternatives to VSVg to improve vector performance in T-cells.

Importantly, clinical-grade viral vectors are a major cost driver in therapeutic cell manufacturing, largely due to the complexity and inefficiencies of vector production and strict quality and safety testing requirements based on their pathogenic origin (31, 32). Strategies that improve viral vector production inefficiencies and scales should help alleviate this as a cost bottleneck of CAR-T and other gene-modified cell therapies.

Non-viral based gene editing technologies are actively being explored as they significantly simplify supply chain constraints and cost burden associated with viral vectors. Transposon/transposase systems, such as piggyBac and Sleeping Beauty are based on co-delivery of the transposase enzyme with a plasmid encoding the therapeutic gene of interest spanned by two inverted terminal repeats (ITRs) that are recognized and targeted by the transposase to promote semi-random genomic integration (33). The use of electroporation to improve plasmid delivery efficiency has enabled clinical exploration of transposon/transposase systems to generate CAR-modified T cells (34–36). As these systems are based on semi-random integration, additional work will help to establish the safety profile of this editing platform.

Targeted gene editing technologies such as ZFN, TALENs, and CRISPR/Cas9 are also being explored for gene modification of adoptive cellular immunotherapy, but to date have mainly been applied to gene knockout of TCR and HLA to create allogeneic or universal T-cells (37) or knocking out immune checkpoint inhibitory pathways such as programmed cell death protein 1 (PD1) (38). The use of CRISPR/Cas9 system to introduce CAR is still limited by technological challenges, primarily related to efficiencies of knocking-in larger genes, although advancements are being made in this regard. CRISPR/Cas9 holds significant promise for a more controlled, targeted gene-editing platform that may increase the safety profile of these therapies.

### Expansion

For most cell therapies, cell expansion is required to reach the clinical dose required. There are several platforms available that enable expansion of the cells. For immunotherapies, the most commonly used systems are static gas permeable culture bags, G-Rex bioreactors, wave-mixed bioreactors, and the Miltenyi Prodigy system, each having their own inherent advantages and disadvantages. There are also several new technology platforms that are being developed for the growth of cellular therapies.

Several companies offer gas permeable bags (GPB) including VueLife (Saint Gobain), Charter Medical and OriGen. GPB are designed to enable a high rate of gas transfer to the cells while maintaining low water permeability enabling culturing of cells in a closed system, unlikely conventional tissue culture flasks, and several groups have demonstrated growth of T cells in these bags (39, 40). Although cost effective to implement initially due to low baseline costs and no requirement to purchase specialized equipment, long term costs may be higher due to lack of in-line analytics and automation.

G-Rex bioreactors (Wilson Wolf) are tissue culture vessels that have a gas-permeable membrane at the base of the vessel and allow for expansion of cells to a high density due to efficient gas exchange at the cell-liquid interface. The G-Rex bioreactors allow expansion of cells from a low seeding density and don't necessary require a media exchange as the design has a large enough reservoir of media to enable culture for 8–10 days. As they mimic the format and handling of tissue culture flasks, they can represent a simple and cost-effective way to initially transfer a process from a preclinical to early clinical setting. However, their lack of automation, integration for process closure, and in-process monitoring capabilities limit their utility in larger scale commercial manufacturing. Due to the static culture environment, G-Rex bioreactors are good at culturing therapies that require feeder cells, such as tumor-inflitrating lymphocytes (TILs) and antigen-specific T cells, as the cell-to-cell contact is not disrupted (41–43).

Wave-mixed bioreactors use the rocking wave motion to enable efficient mixing and oxygen transfer within the reactor in a low shear side-to-side motion (44) and have become the most common platform for commercial scale-up of T-cell immunotherapies. There are several wave-mixed bioreactors on the market including the SmartRocker (Finesse), Allegro (Pall), Biostat RM (Sartorius), and Xuri Cell Expansion System (GE Healthcare), among others. Due to the lower volume specifications required for patient-specific immunotherapies (often no more than ~1 L of culture medium) the Xuri Cell Expansion System and the Biostat RM are widely used. Wave-mixed bioreactors are functionally closed, and reduce the amount of manual labor in the cell expansion phase of the culture due to their built-in automation features. Single-use sensors enable the monitoring of several parameters including pH, dissolved oxygen (DO), as well as pumps that enable perfusion to intensify the culture of cells to a higher cell density than possible with conventional fed-batch or medium replacement regimens. Due to the design of the systems there is usually a minimum starting volume (~300mL in a 1L working volume bag, for example) in these bioreactors thus a limitation of these systems is that they require an initial seed train for the start of the cell expansion before the culture reaches the required volume to be transferred into the wave-mixed single-use bag.

The CliniMACS Prodigy (Miltenyi Biotec) is an all-in-one solution that not only enables immune cell expansion, but several other steps within the immunotherapy workflow including cell selection through magnetic separation, cell washing, and final product formulation in a closed system. The CliniMACS Prodigy supports a culture volume of up to 400 mL (300 mL working volume). Although using the CliniMACS Prodigy has many benefits in terms of ease-of-use, and reduction in contamination risk due to its amenability for closed separation and expansion, the use of a single system throughout the manufacturing process may not be as cost effective as having individual unit operations. Specifically, with the all-in-one approach, the entire instrument is occupied even when a single unit operation is being performed, whereas freeing up individual device unit operations for additional patient material may be more desirable from a manufacturing standpoint.

There are also several alternative technologies that can potentially be used for culture expansion of immunotherapies. Stirred tank reactors (STRs) are commonly used in monoclonal antibody production and have a scale ranging from hundreds of milliliters (e.g., spinner flasks) to thousands of liters. Culturing T cells in an STR could be useful in an allogenic setting, where scale-up will be more important (vs. scale out with patient-specific therapies). To this end, GE Healthcare has developed the Xcellerex XDR line ranging in volume from 10 to 2,000 L in single-use format. PBS Biotech has developed a new series of bioreactors that use a vertical wheel to enable homogeneous mixing with less shear force compared to standard STRs. The PBS systems may be an interesting option for adherent cells cultured on microcarriers or in aggregates and their use with immunotherapies could be worth exploring given the novel design, efficient mixing, amenability for single-use, low shear, compatibility with DO and pH probes, and scales ranging from 20 mL to 500 L suitable for either patient-specific or allogeneic workflows. Other technologies include Octane Biotech's Cocoon™ Bioreactor, a non-agitated, closed, all-in-one system enabling fully automated seeding, expansion, perfusion, digesting/harvesting, concentration, washing and formulation within a single chamber. The single-use cassettes are amenable for patient-specific use and can monitor pH, DO, control temperature and gases, and expand either adherent or suspension-grown cells. The Quantum™ Cell Expansion System from TerumoBCT is another closed, automated system based on a hollow fiber bioreactor which can culture both adherent or suspension cells. Each single-use cartridge comprises over 11,500 hollow fibers to generate a surface area of 2.1 m^2^, making them an intriguing option for use in cell therapy manufacturing (45).

### In-process analytics

As the field of immunotherapy progresses from academic workflows to clinical manufacturing, the need for continuous measurement of CQAs will be the next frontier; this broadly describes the field of in-line process analytical technologies (PAT). While process sensors to monitor DO, pH, and temperature are commonplace in bioreactor systems, off-line, manual sampling is routinely performed to capture other process readouts such as cell density, viability, and metabolites. Systems such as the SegFlow (Flownamics) can enable closed and automated sampling and integration with relevant analytical platforms for cell counting (e.g., ViCell) or metabolite analysis (e.g., CEDEX BioHT, FLEX2), enabling scheduled monitoring of up to 8 reactors and sample delivery to 4 analytical instruments. These can moreover be setup with programmable feedback loops to adjust feed regimes in response to measured values, thus removing the operator from the equation. Limitations still exist in terms of minimum sample/hold-up volumes, distances between equipment, and communication between systems from different vendors.

Ideally, these analytical technologies will be integrated into the culture vessels to provide real-time, in-line monitoring. Conversion of existing autoclavable probe technologies for biomass (Aber, Hamilton), glucose, and lactate to single-use disposable formats will further automate and de-risk in-process sampling. For example, bio-capacitance probes have been integrated into the Sartorius' BIOSART RM rocking bioreactor bags. The Xuri W25 Cell Expansion System (GE Healthcare) rocking bioreactor collects data from single-use DO and pH probes, which can be used as surrogate measures of VCD and enable informed decisions on perfusion and DO control without the need for sampling. Alternatively, the iLine-F in-line holographic imaging system (Ovizio Imaging Systems) enables real time readouts of viability, cell density, and potentially relevant morphological parameters.

Other promising sensor platforms include Raman for chemical composition/cell cycle state (46); radio frequency and microwave sensors for detection of biomass and other analytes (47); and near infrared (nIR) spectroscopy for biomass detection (48). Importantly, the raw data collected from these various sensing technologies will need to be processed using statistical tools such as principal component analysis (PCA) to draw correlations between surrogate measures and actual CQAs they are meant to quantify.

The long term goal or “holy grail” would be to use PAT data to inform on nearly all process decisions in real-time, or even predictively. This can include decision points on LVV transduction, when to initiate perfusion based on DO, when to harvest based on VCD, or even QC release based on minimum viability or endotoxin level. Ideally, such technologies will evolve to measure surface marker expression of key phenotypic markers such as CD3, CD4, CD8 for T-cells and CD56 for NK cells, for example, or even more specific metrics around activation state (e.g., CD25 expression for T-cells), cytokine release, and even CAR expression to inform on transduction efficiency. The latter could eliminate the need for sampling for flow cytometry altogether. A great deal of work will be needed to develop both reliable potency assays to which these readouts can be correlated, as well as low cost, single-use sensing technologies capable of these feats.

### Downstream processing

Downstream cellular processing encompasses the harvest, collection, wash, and formulation of the cell product. Depending on the specifics of the upstream process or target product composition, additional purification steps such as de-beading or cell selection may be required. In general, the expanded cell product is collected from the culture vessel, concentrated and washed to replace culture media with formulation buffer, and filled into multiple cryobags for the patient dose as well as QC samples and retains.

Ultimately, the process will need to be designed to meet release criteria specifications related to input volume/concentration, final volume/concentration, viability, and removal of culture residuals. The field has evolved from multiple conventional batch centrifugation wash cycles toward platforms that are closed, automated and single-use. Many groups re-purpose upstream cell processing systems like the Sepax II (GE Healthcare), CliniMACS Plus (Miltenyi Biotec), Elutra (Terumo BCT), Cobe 2991 (Terumo BCT), or CellSaver-5 (Haemonetics) to perform cell concentration and wash. Several dedicated downstream platforms have also hit the market this decade including the Sefia (GE Healthcare), kSep 400 (Sartorius Stedim), Lovo (Fresenius Kabi), and SynGenX-Wash (Syngen).

While many of the systems listed above are functionally closed, closed integration with neighboring unit operations remains a challenge, particularly when mating consumables from different vendors. Formulation compatibility can also pose hurdles, particularly if the final formulation is a dimethyl sulfoxide (DMSO)-containing cryoprotectant, which is currently incompatible with sterile weldable flexible PVC tubing and may be cytotoxic with prolonged exposure (49, 50). This often requires users to perform an additional step of DMSO addition to a basal buffer or medium, either manually or via a dedicated device like the SmartMax (GE Healthcare), which can perform cryoprotectant formulation under automated, closed, and temperature controlled conditions. As manufacturing demand heightens, considerations such as processing time and automated integration will be increasingly important factors during scale-out.

### Cryopreservation and thaw

Cell therapy manufacturing is now exploring the use of automated and quantitative tools for cryopreservation and thaw. Programmable controlled rate freezers (CRFs) employing liquid nitrogen as a refrigerant are already popular among cell therapy manufacturers (51, 52) giving greater control and customizability of cooling rates as well as ample volume for freezing of bags and vials alike. Novel freezing systems that do not require liquid nitrogen have recently emerged from Asymptote (recently acquired by GE Healthcare), such as the Asymptote VIA Quad, Duo, and Research Freezers. Like CRFs, these are automated, programmable freezers, but are also amenable for use in GMP cleanroom facilities where the use of liquid nitrogen poses additional risks in terms of contamination and air quality (53). While DMSO has dominated as the cryopreservation agent (CPA) of choice for decades for mammalian cells, it may soon be entirely supplanted by DMSO-free options due to their lower risk profile, enhanced patient experience, better compatibility with weldable tubing (discussed above), and possibility of eliminating washing steps prior to patient infusion. Table 1 below summarizes some DMSO-based and DMSO-free offerings used by cell therapy manufacturers, all of which have been shown by our group to be suitable for use with immune cell populations with high post-thaw recovery and viability.

**Table 1:** DMSO-free and DMSO-containing cryoprotectants.

**Company**	**Cryoprotectants**	**DMSO %**	**ACF/XF**	**GMP**
Biolife solutions	CryoStor 10	10%	ACF and XF	GMP
Akron	P24 CryoNovo	DMSO-Free	ACF and XF	GMP-available 2018
Akron	M26 CryoNovo	DMSO-Free	ACF and XF	GMP-available 2018
Irvine scientific	Prime-XV FreezIS	DMSO-Free	ACF and XF	GMP
GE HyClone	Cryopreservation media	7%	ACF and XF	GMP
Takara/Amsbio	Stem CellBanker	DMSO-FREE	ACF and XF	GMP

GE's Asymptote and other companies (such as Medcision and Sarstedt) have also developed automated dry-thawing devices which can eliminate the risks associated with manual thawing in a water bath, such as water-borne contaminants, subjectivity in thawing time, and operator-to-operator variability. The Asymptote VIA Thaw SC2 and Biocision ThawSTAR are designed to thaw vials, while the Asymptote VIA Thaw CB1000, Medcision ThawCB, and Sarstedt Sahara system are capable of thawing bags containing cord blood and other immune cell populations. Overall, it is anticipated that early adoption of these systems and reagents will ensure better process reproducibility and product stability prior to patient administration.

## Future directions

As demand for patient-specific immunotherapies increases, novel solutions will undoubtedly emerge to address patient needs. Further automating and controlling cell therapy manufacturing processes not only reduces patient risk and leads to better product robustness, but also lowers costs, and thus will enable better patient access. Moreover, novel generation CAR constructs may address both the safety and cost issues simultaneously—for example, new 4th generation CARs (so-called “TRUCK” or “armored CAR” T-cells) may be able to secrete therapeutic proteins or drug payloads, and may incorporate suicide genes to provide better control over cytokine release syndrome, thereby reducing cell dosing requirements (and thus, cost) while increasing patient safety. Viral gene editing strategies may migrate toward non-viral methods, which may simplify supply chain logistics and costs.

As improvements in reactor capacity, single-use consumables, up and downstream integration, analytics, sensing technologies, culture media, equipment footprint, and customization/automation arise, so too will costs undoubtedly drop, as will patient access to therapies.

Importantly, incorporation of new process technologies is not a trivial exercise, particularly in later stages of clinical development or commercial manufacturing, since comparability to the clinically tested product must be performed, creating a high barrier for manufacturing process changes. Newer manufacturing technologies and platforms will have their greatest impact on early stage clinical products, where they can be designed and validated in IND submissions for Chemistry, Manufacturing and Control (CMC) documentation.

While most autologous cell therapies are currently centrally manufactured, a decentralized approach may well enable global dissemination of cell therapies on a larger scale. To ensure this vision of cell therapy manufacturing becomes a reality, equipment, and reagent manufacturers, along with clinicians, research institutions, and CDMOs, will need to work together in a unified effort to democratize access to these highly efficacious treatments.

## Author contributions

RKI prepared the following sections: Abstract, Introduction, Reagents, In-Process Analytics, Cryopreservation and Thaw, Future Directions, Table 1. PB prepared the following sections: Selection, Activation, Expansion. HK prepared the following sections: Transduction, Downstream Processing, References, Figure 1. AD-T: reviewed the entire manuscript and provided feedback on all sections. RKI, PB, and HK: all edited the manuscript.

### Conflict of interest statement

The authors declare that the research was conducted in the absence of any commercial or financial relationships that could be construed as a potential conflict of interest. The reviewer AH and handling Editor declared their shared affiliation.
